# Evaluation of the clinical impact of bone marrow cultures in current medical practice

**DOI:** 10.1038/s41598-022-14059-3

**Published:** 2022-06-11

**Authors:** Gal Sharvit, Daniel Schwartz, Gabriel Heering, Alexander Shulman, Abraham Avigdor, Galia Rahav, Amos Toren, Arnon Nagler, Jonathan Canaani

**Affiliations:** 1 Division of Hematology, Chaim Sheba Medical Center, Faculty of Medicine, Tel Aviv University, Tel Hashomer, Derech Sheba 2, 52621 Ramat Gan, Israel; 2 MetroHealth Medical Center, Case Western Reserve University, 2500 Metrohealth Dr, Cleveland, OH 44109 USA; 3 Westchester Medical Center, 100 Woods Rd, Valhalla, NY 10595 USA; 4 The Infectious Diseases Unit, Chaim Sheba Medical Center, Faculty of Medicine, Tel Aviv University, Tel Hashomer, Derech Sheba 2, 52621 Ramat Gan, Israel; 5 Pediatric Hemato-Oncology Division, Chaim Sheba Medical Center, Faculty of Medicine, Tel Aviv University, Tel Hashomer, Derech Sheba 2, 52621 Ramat Gan, Israel

**Keywords:** Diagnosis, Laboratory techniques and procedures

## Abstract

The clinical yield and benefit of performing bone marrow cultures for various clinical indications has been challenged and their clinical necessity remains debatable. We sought to assess the clinical yield and benefit of performing routine bone marrow cultures and determine whether various clinical, laboratory, and imaging parameters were predictive of a diagnostic bone marrow culture. This was a single center retrospective analysis of all patients who underwent a bone marrow study comprising bone marrow cultures from January 1, 2012, through March 1, 2018. Baseline clinical data were extracted from the institution's electronic medical records system. The analyzed cohort consisted of 139 patients with a median age of 46 years (range 4 months to 85 years). The most common indication for a bone marrow study was workup of a fever of unknown origin (105 patients, 76%) while investigation for infection in immunocompromised patients accounted for 22 cases (16%) and suspected tuberculosis was the reason for acquisition of bone marrow cultures in 6 patients (4%). Only 3 patients had positive bone marrow cultures, yielding in 2 patients a diagnosis of Mycobacterium avium and in one patient a microbiologically unclassifiable fungal infection. A univariate analysis revealed that mean age, hemoglobin level, platelet count, c-reactive protein levels, gender, indication for bone marrow study, yield of blood cultures, and contribution of imaging studies and bone marrow pathology results were not significantly different between patients with diagnostic and non-diagnostic bone marrow cultures. Mean white blood cell count was found to be significantly lower in patients with diagnostic bone marrow cultures (2.4 × 10^3^/µL versus 8.7 × 10^3^/µL; *P* = 0.038). We conclude that for most patients, performance of bone marrow cultures holds limited clinical value.

## Introduction

Whereas bone marrow studies form a key element of the routine workup and investigation of patients presenting with a clinical picture suspicious for an underlying malignant or benign hematologic pathology, the clinical benefit of performing bone marrow studies for extended indications such as workup of fever of unknown origin (FUO) is less clear^1–3^. In particular, the practice of performing bone marrow cultures has been challenged and its clinical necessity and yield remain questionable^4,5^. Indeed, earlier studies focusing on immunosuppressed patients aiming at identification of mycobacterial and fungal infections have for the most part resulted in low microbiologic recovery rates^6–11^. In contrast, previous studies in patients suspected of having Kala azar have shown bone marrow cultures to be a significant adjunct in definitive diagnosis of parasitic disease^12^. By the same token, earlier data indicates bone marrow cultures are also a sensitive diagnostic modality for diagnosis of typhoid fever^13^. Thus, at present there is clinical uncertainty pertaining to the diagnostic role bone marrow cultures hold in current medical practice. In this study, we paired bone marrow microbiologic data with comprehensive clinical annotation in a large cohort of patients who had bone marrow cultures performed as part of their medical workup. Our findings suggest that bone marrow cultures have low diagnostic yield and limited practical benefit in most clinical scenarios.

## Results

### Demographic characteristics

We identified 139 patients who were admitted to our medical center in 2012–2018 and had bone marrow cultures performed as part of their diagnostic workup. Median patient age was 45 years (range 4 months to 85 years). As shown in Table 1, the median WBC count at diagnosis was 6.9 × 10^9^/l (range 0.5–36) and the median platelet count was 212 × 10^9^/l (range 5–1280). Twenty-three patients (17%) had a previously diagnosed hematologic malignancy whereas 3 patients (2%) had a diagnosis of a solid malignancy. The most common indication for a bone marrow study was workup of a fever of unknown origin (FUO) (105 patients, 76%) while investigation for infection in immunocompromised patients accounted for 22 cases (16%) and suspected tuberculosis was the reason for acquisition of bone marrow cultures in 6 patients (4%).

**Table 1:** Baseline characteristics of 139 patients who had bone marrow cultures performed in 2012–2018.

Clinical parameter	
Age in year, median (range)	46 (0.3–85)
**Gender, n (%)**	
Male	86 (62)
Female	53 (38)
**Baseline laboratory parameters, median (range)**	
Hemoglobin (g/dL)	9.7 (4.6–15.3)
WBC (× 10^3^/µL)	6.9 (0.5–36)
Platelets (× 10^3^/µL)	212 (5–1280)
CRP (mg/L)	88 (0.1–396)
HIV positive, n (%)	11 (8)
TB positive, n (%)	0
**Medical background, n (%)**	
Baseline hematological malignancy	23 (17)
Baseline solid malignancy	3 (2)
No malignancy	104 (76)
Bone marrow biopsy revealed new malignancy	6 (4)
Other biopsy revealed new malignancy	1 (1)
Missing	2
**Indication for bone marrow culture, n (%)**	
Workup of FUO	105 (76)
Suspected infection in immunocompromised patient	22 (16)
Suspected tuberculosis	6 (4)
Other	5 (4)
Missing	1

### Contribution of ancillary studies to final diagnosis

Table 2 outlines the results of the various diagnostic modalities employed during the medical workup and their diagnostic contribution to reaching a final diagnosis. Of 139 patients who underwent bone marrow examinations with culture studies, only 3 patients had positive bone marrow cultures yielding in 2 patients a diagnosis of Mycobacterium avium and in one patient a microbiologically unclassifiable fungal infection. None of these patients were found to concurrently harbor the bone marrow culture pathogen in other tissues. Virology studies, blood cultures, and urine cultures were contributory to the final diagnosis in 12 (9%), 6 (4%), and 2 (1%) patients, respectively. Bone marrow pathology results resulted in a diagnosis of a hematologic malignancy in 15 patients (11%) and a solid malignancy in one patient (1%). Chest X-rays were indicative for an infectious process in 11 patients (10%) while CT or PET/CT scans demonstrated imaging findings suspicious for an infection in 12 patients (14%) and suspicious lymphadenopathy in 25 patients (28%).

**Table 2:** Contribution of microbiologic, imaging, and bone marrow biopsy results to final diagnosis.

Clinical parameter	
Blood cultures contributory to final diagnosis, n (%)	6 (4)
Urine cultures contributory to final diagnosis, n (%)	2 (1)
Virology studies contributory to final diagnosis, n (%)	12 (9)
**Bone marrow cultures results**	
Non diagnostic	136 (98)
Diagnostic	3 (2)
**Bone marrow biopsy results contributory to final diagnosis**	
No	97 (70)
Hematologic malignancy	15 (11)
Other diagnosis	5 (3)
Solid malignancy	1 (1)
Missing	21 (15)
**Chest X-ray contributory to final diagnosis, n (%)**	
No	98 (90)
Suspicious for infection	11 (10)
Not performed	30
**CT or PET/CT scan contributory to final diagnosis, n (%)**	
No	52 (58)
Suspicious for infection	12 (14)
Suspicious lymphadenopathy	25 (28)
Not performed	50

### Final diagnosis

Table 3 outlines the final diagnosis reached for patients who underwent bone marrow cultures as part of their diagnostic workup. Thirty patients (21.6%) were diagnosed with an infection which included 4 cases of mycobacterial infection, 3 of whom were with *Mycobacterium avium* and one patient with *Mycobacterium kansasii*. Leishmaniasis and EBV infections were the second most common infectious etiologies in this patient cohort, seen in 3 patients in each group. Two patients were diagnosed with *Coxiella burnetti* infection whereas nocardial and *Bartonella henselae* infections were seen each in one patient. Autoimmune and rheumatologic diseases were the most common medical conditions diagnosed in this cohort seen in 40 patients (28.8%). Hemophagocytic lymphohistiocytosis (HLH) was the most common autoimmune condition seen (5 patients) while Juvenile rheumatoid arthritis and Adult-onset Still's disease were both seen in 3 patients each. In 18 patients (12.9%) malignancy accounted for the patients' clinical presentation with lymphoma being the leading neoplastic etiology, seen in 10 patients (3 with peripheral T-cell lymphoma, 3 with Hodgkin lymphoma, 2 with diffuse large B-cell lymphoma, and follicular lymphoma and Burkitt lymphoma seen each in one patient). In 51 patients no final diagnosis was reached.

**Table 3:** Final diagnoses of 139 patients who underwent bone marrow culture testing.

Clinical parameter	
Hemophagocytic lymphohistiocytosis	5
Mycobacterial infection	4
Leishmaniasis	3
Juvenile rheumatoid arthritis	3
Peripheral T-cell lymphoma	3
Adult-onset Still's disease	3
Hodgkin lymphoma	3
Inflammatory bowel disease	3
EBV infection	3
Common variable immunodeficiency	2
Solid tumor, undefined	2
Polymyalgia rheumatica	2
Urinary tract infection	2
Pericarditis	2
Vasculitis, unspecified	2
Diffuse large B-cell lymphoma	2
Autoimmune/rheumatologic disease, undefined	2
Rhematologic disorder, undefined	2
Fungal infection	2
Drug induced fever	2
Spinal fracture	1
Spinal abscess	1
Systemic lupus erythematosus	1
Sinusitis	1
Hairy cell leukemia	1
serum sickness	1
Sarcoidosis	1
Respiratory infection	1
Rheumatoid arthritis	1
Q fever—*Coxiella burnetti*	2
Poorly differentiated adenocarcinoma	1
Vasculitis, DADA 2	1
Progressive multifocal leukoencephalopathy	1
PFAPA syndrome	1
Otitis media	1
Nocardial infection	1
Myelodysplastic syndrome	1
Liver abscess	1
Infectious endocarditis	1
HIV-new diagnosis	1
Graft-versus-host disease	1
Granulomatous hepatitis	1
Giant cell arteritis	1
FUO secondary to myeloma	1
Follicular lymphoma	1
CMV	1
Chronic lymphocytic leukemia	1
Chronic osteomyelitis	1
Castleman's disease	1
Burkitt lymphoma	1
Bartonella henselae	1
Bacterial infection	1
Autoimmune hepatitis	1
Acute lymphoblastic leukemia	1
Adenovirus infection	1
No diagnosis reached	51

### Analysis of clinical factors predictive of a diagnostic bone marrow culture

In order to determine whether any of the baseline clinical, laboratory, imaging, and pathologic data would be predictive of a diagnostic bone marrow culture we proceeded to perform univariate analyses comparing these parameters between the group of patients with diagnostic bone marrow cultures with those with non-diagnostic bone marrow cultures. As summarized in the statistical analysis shown in Table 4 using T-Test and Levene's Test for Equality of Variances, mean age, hemoglobin level, platelet count, and CRP levels were not significantly different between patients with diagnostic bone marrow cultures and those with non-diagnostic bone marrow cultures. However, the mean white blood cell count was found to be significantly lower in patients with diagnostic bone marrow cultures compared with those patients with non-diagnostic bone marrow cultures (2.4 × 10^3^/µL versus 8.7 × 10^3^/µL; *P* = 0.038). In terms of categorical variables, assessment with Pearson's chi-square test, revealed that there was no statistically significant difference between patients with diagnostic bone marrow cultures and patients with non-diagnostic bone marrow cultures in terms of gender, indication for bone marrow study, yield of blood cultures, and contribution of imaging studies and bone marrow pathology results to final diagnosis (Table 5). However, all the patients with diagnostic bone marrow cultures had a final diagnosis of infection compared with 32% of patients with non-diagnostic bone marrow cultures (*P* = 0.049).

**Table 4:** Analysis of quantitative factors predictive of a diagnostic bone marrow culture.

Clinical parameter	Diagnostic BM culture	Non-diagnostic BM culture	*P*
Age (year), mean (SD)	31.6 (10.9)	41.9 (26.6)	0.5
WBC (× 10^3^/µL), mean (SD)	2.4 (2.6)	8.7 (7.3)	0.038
Hemoglobin (g/dL), mean (SD)	8.4 (1.1)	9.9 (1.7)	0.15
Platelets (× 10^3^/µL), mean (SD)	143 (101)	249 (193)	0.34
CRP (mg/L), mean (SD)	133.6 (53.5)	111.3 (98.1)	0.69

**Table 5:** Analysis of factors predictive of a diagnostic bone marrow culture.

Clinical parameter	Diagnostic BM culture	Non-diagnostic BM culture	*P*
**Gender, n (%)**			1
Male	2 (67)	84 (62)	
Female	1 (33)	52 (38)	
**Indication for bone marrow study**			0.087
Workup of FUO	2 (67)	103 (76)	
Suspected infection in immunocompromised patient	0	22 (16)	
Suspected tuberculosis	1 (33)	5 (4)	
Other	0	5 (4)	
**Blood cultures contributory to final diagnosis**			1
No	3 (100)	127 (96)	
Yes	0	6 (4)	
**Urine cultures contributory to final diagnosis**			1
No	3 (100)	106 (98)	
Yes	0	2 (2)	
**Virology studies contributory to final diagnosis**			0.24
No	2 (67)	124 (92)	
Yes	1 (33)	11 (8)	
**CT or PET/CT scan contributory to final diagnosis**			0.16
No	0	52 (60)	
Suspicious for infection	1 (50)	11 (12)	
Suspicious lymphadenopathy	1 (50)	24 (28)	
**HIV status**			0.22
Negative	2 (67)	126 (93)	
Positive	1 (33)	10 (7)	
**Bone marrow biopsy results contributory to final diagnosis**			0.94
No	1 (33)	22 (16)	
Hematologic malignancy	0	3 (2)	
Other diagnosis	2 (67)	102 (76)	
Missing	0	6 (5)	
Solid malignancy	0	1 (1)	
**Final diagnosis**			0.049
Infection	3 (100)	27 (32)	
Rheumatologic/autoimmune disorder	0	40 (47)	
Malignancy	0	18 (21)	

## Discussion

Over the past four decades the use of bone marrow cultures has been mostly delegated to the investigation of infections in immunocompromised patients with the aim of increasing the diagnostic yield afforded by standard microbiological studies. However, hitherto the specific patient segment deriving the most clinical benefit from use of bone marrow cultures has not been defined. The outcomes of interest in the present study were first to delineate our experience and the clinical yield with bone marrow cultures in a contemporary cohort of patients, and second to identify potential clinical parameters predictive of a diagnostic bone marrow culture. Our findings suggest that in most clinical scenarios encountered in current medical practice, routine performance of bone marrow cultures is of limited clinical value.

Many of the previous studies addressing the use of bone marrow cultures have focused on the HIV patient subset with most investigators suggesting that bone marrow cultures offered a comparable or inferior diagnostic sensitivity to routine blood cultures for detection of mycobacterial and fungal infections^4,9,11,14^. In agreement with these data, we observed that of 139 patients who had bone marrow cultures performed, only three patients, one of whom was HIV positive, had positive bone marrow cultures yielding in 2 patients a diagnosis of Mycobacterium avium and in one patient a diagnosis of a fungal infection. Thus, our findings lend further credence to what Riley et al. previously suggested, namely that bone marrow cultures should be reserved for severely immunosuppressed patients and should not be routinely employed in immunocompetent patients^7^.

As previous studies aimed at delineating the role of bone marrow studies in the workup of FUO and attempted to identify patients with a higher likelihood of deriving clinical benefit from bone marrow studies in this setting^1–3^, we also sought to determine whether we could identify predictive parameters for a diagnostic bone marrow culture. Assessing a multitude of baseline demographic, clinical, laboratory, and imaging parameters we found that the only parameter predictive of a diagnostic bone marrow culture was a lower mean white blood cell count. Owing to the small number of patients with diagnostic bone marrow cultures we believe prudent interpretation of this association is warranted.

All things considered, our study reaffirms earlier observations pertaining to the current role and expected yield of bone marrow cultures in contemporary medical practice. Our data would suggest that exclusive of the setting of severely immunocompromised patients with a high clinical suspicion for occult mycobacterial and fungal infection, bone marrow cultures are generally of low yield and their routine use should be reconsidered.

There are some aspects of our study that warrant comment. The primary limitations of the study consist of its retrospective design and a patient population from a single tertiary referral center with the incurrent potential referral bias. Further, the small number of diagnostic bone marrow cultures restrict the capacity for definitive conclusions and mandate cautious interpretation of our analysis.

The results of this analysis suggest that bone marrow cultures hold limited value in most clinical scenarios. The implications of these findings for clinical practice are that for most patients, with the possible exception of severely immunocompromised patients, the expected yield of bone marrow cultures is low and consequently should not be routinely performed.

## Methods

### Study cohort

We reviewed the electronic medical records of 139 consecutive patients from the Chaim Sheba Medical Center, Tel-Hashomer, from January 1, 2012, through March 1, 2018, who underwent a bone marrow study comprising bone marrow cultures. Data on comorbidities, baseline laboratory parameters, and indication for performing a bone marrow culture were extracted from the Sheba electronic medical records system. In addition, we collected antimicrobial and imaging information performed during the index hospital admission during which the bone marrow cultures were obtained. We defined contributory microbiology studies (i.e., blood and urine cultures, virology studies) as those studies with microbiological isolates which were assessed by the treating medical team to be the principal etiology for the patients' medical condition. Likewise, the results of chest X-rays, computed tomography (CT), and Positron emission tomography-computed tomography (PET/CT) were evaluated by the first author and senior author of the study (GS, JC) and determined whether they were contributory to the final diagnosis. Bone marrow biopsies were performed by puncture of the posterior iliac crest using a Jamshidi needle. Sections were sent in formalin solution for histologic processing and stained with standard hematoxylin–eosin stains. Immunochemistry staining was performed if a malignant neoplasm was identified during initial pathologic review. Microbiology studies were undertaken by bedside inoculation of bone marrow aspirates in bacterial (BACTEC FX; Becton Dickinson, Franklin Lakes, New Jersey) and mycobacterial culture medium (BACTEC MGIT 960, Becton Dickinson, Franklin Lakes, NJ).

The Institutional Review Board of the Chaim Sheba Medical Center approved this study. All data were deidentified.

### Statistical analysis

T-test and the Mann–Whitney tests were used to compare the distributions of quantitative continuous variables. Nominal data were compared using Fisher's exact test or Pearson's chi-square test. To identify clinical parameters associated with a diagnostic bone marrow culture, a univariate analysis was performed, using demographic and medical parameters as well as all clinical, laboratory, microbiological, and imaging data obtained during the index hospital admission. All reported P values were based on 2-sided tests, and P ≤ 0.05 was considered statistically significant. Analyses were performed using SPSS 25.0 (SPSS Inc, Chicago, IL, USA).

### Informed consent

Owing to the retrospective and anonymized nature of this retrospective chart review study, a waiver was granted by the Sheba Medical Center Institutional Review Board.

### Research involving human participants

This retrospective chart review study involving human participants was in accordance with the ethical standards of the institutional and national research committee and with the 1964 Helsinki Declaration and its later amendments or comparable ethical standards. The Human Investigation Committee (IRB) of the Chaim Sheba Medical Center approved this study.

## Data Availability

The dataset generated during and analyzed in the study are available from the corresponding author on reasonable request.

## References

[1] Hot A (2009). Yield of bone marrow examination in diagnosing the source of fever of unknown origin. Arch. Intern. Med..

[2] Ben-Baruch S (2012). Predictive parameters for a diagnostic bone marrow biopsy specimen in the work-up of fever of unknown origin. Mayo Clin. Proc..

[3] Wang HY (2014). A "bone marrow score" for predicting hematological disease in immunocompetent patients with fevers of unknown origin. Medicine.

[4] Volk EE, Miller ML, Kirkley BA, Washington JA (1998). The diagnostic usefulness of bone marrow cultures in patients with fever of unknown origin. Am. J. Clin. Pathol..

[5] Arya A, Naithani R (2017). Futility of performing bone marrow cultures in pyrexia of unknown origin. Indian J. Hematol. Blood Transfus..

[6] Bishburg E, Eng RH, Smith SM, Kapila R (1986). Yield of bone marrow culture in the diagnosis of infectious diseases in patients with acquired immunodeficiency syndrome. J. Clin. Microbiol..

[7] Riley UB, Crawford S, Barrett SP, Abdalla SH (1995). Detection of mycobacteria in bone marrow biopsy specimens taken to investigate pyrexia of unknown origin. J. Clin. Pathol..

[8] Kilby JM (1998). The yield of bone marrow biopsy and culture compared with blood culture in the evaluation of HIV-infected patients for mycobacterial and fungal infections. Am. J. Med..

[9] Hussong J, Peterson LR, Warren JR, Peterson LC (1998). Detecting disseminated *Mycobacterium avium* complex infections in HIV-positive patients. The usefulness of bone marrow trephine biopsy specimens, aspirate cultures, and blood cultures. Am. J. Clin. Pathol..

[10] Talbot EA, Reller LB, Frothingham R (1999). Bone marrow cultures for the diagnosis of mycobacterial and fungal infections in patients infected with the human immunodeficiency virus. Int. J. Tuberc. Lung Dis..

[11] Akpek G, Lee SM, Gagnon DR, Cooley TP, Wright DG (2001). Bone marrow aspiration, biopsy, and culture in the evaluation of HIV-infected patients for invasive mycobacteria and histoplasma infections. Am. J. Hematol..

[12] Sinha R, Datta U, Sehgal S (1993). Importance of bone marrow culture for diagnosis of Kala azar. Scand. J. Infect. Dis..

[13] Akoh JA (1991). Relative sensitivity of blood and bone marrow cultures in typhoid fever. Trop. Doct..

[14] Pacios E (2004). Evaluation of bone marrow and blood cultures for the recovery of mycobacteria in the diagnosis of disseminated mycobacterial infections. Clin. Microbiol. Infect..

